# Self-collected genital swabs compared with cervicovaginal lavage for measuring HIV-1 and HSV-2 and the effect of acyclovir on viral shedding

**DOI:** 10.1177/0956462416650123

**Published:** 2016-07-10

**Authors:** Janet M McNicholl, Wanna Leelawiwat, Sara Whitehead, Debra L Hanson, Tammy Evans-Strickfaden, Chen Y Cheng, Wannee Chonwattana, Famui Mueanpai, Chonticha Kittinunvorakoon, Lauri Markowitz, Eileen F Dunne

**Affiliations:** 1 Division of HIV/AIDS Prevention, Centers for Disease Control and Prevention, GA, USA; 2 The Thai Ministry of Public Health, U.S. Centers for Disease Control and Prevention Collaboration, Nonthaburi, Thailand; 3 Division of STD Prevention, Centers for Disease Control and Prevention, GA, USA

**Keywords:** Virus, polymerase chain reaction, sampling, sexually transmitted infection, ano-genital, transmission, self-collection

## Abstract

HIV-1 and HSV-2 are frequent genital co-infections in women. To determine how self-collected genital swabs compare to provider-collected cervicovaginal lavage, paired self-collected genital swabs and cervicovaginal lavage from women co-infected with HIV-1 and HSV-2 were evaluated. Women were in an acyclovir clinical trial and their samples were tested for HIV-1 RNA (361 samples) and HSV-2 DNA (378 samples). Virus shedding, quantity and acyclovir effect were compared. HIV-1 and HSV-2 were more frequently detected in self-collected genital swabs: 74.5% of self-collected genital swabs and 63.6% of cervicovaginal lavage had detectable HIV-1 (p ≤ 0.001, Fisher’s exact test) and 29.7% of self-collected genital swabs and 19.3% of cervicovaginal lavage had detectable HSV-2 (p ≤ 0.001) in the placebo month. Cervicovaginal lavage and self-collected genital swabs virus levels were correlated (Spearman’s rho, 0.68 for HIV; 0.61 for HSV-2) and self-collected genital swabs levels were generally higher. In multivariate modeling, self-collected genital swabs and cervicovaginal lavage could equally detect the virus-suppressive effect of acyclovir: for HIV-1, proportional odds ratios were 0.42 and 0.47 and for HSV-2, they were 0.10 and 0.03 for self-collected genital swabs and cervicovaginal lavage, respectively. Self-collected genital swabs should be considered for detection and measurement of HIV-1 and HSV-2 in clinical trials and other studies as they are a sensitive method to detect virus and can be collected in the home with frequent sampling.

## Introduction

HIV-1 transmission is strongly associated with genital HIV-1 shedding^1,2^ and many efforts to reduce transmission focus on reducing genital shedding. Treatment of HIV-infected persons with anti-retroviral drugs reduces viral burden, genital shedding, and HIV-1 transmission.^3^ However, efforts to reduce HIV-1 transmission by targeting HSV-2 have so far failed,^4^ even though HSV-2 is associated with HIV-1 acquisition and transmission (reviewed in Barnabas and Celum^5^) and anti-HSV-2 treatment reduces HIV-1 shedding.^6–9^

Detection and measurement of HIV-1 and HSV-2 at mucosal sites is critical to understanding factors that affect their shedding. Methods that sample genital sites frequently, without requiring visits to a provider, such as self-obtained vaginal samples,^10^ are particularly useful, since genital HIV-1 and HSV-2 shedding varies in relation to many factors^11,12^ (reviewed in Barnabas and Celum^5^).

Cervicovaginal lavage (CVL), cervical and endocervical swabs have been used as the primary sampling methods for HIV-1 detection and quantitation in clinical trials.^10,13–17^ Assaying CVL showed the HIV-suppressive effect of acyclovir in HIV-1 and HSV-2 co-infected women in Thailand.^8^ This sample required a weekly clinic visit and collection by a physician or other trained provider. During the trial, self-collected genital swabs (SCS) were also obtained^18^ to allow comparison with CVL. Although CVL have the advantage of a larger sample volume than swabs, some studies have shown equal or higher HIV-1 detection rates with cervical or endocervical swabs.^13,14^ Wicks, sponges, and self-collected vaginal tampons have also been used for HIV-1 detection.^10,16,17^ While self-collected samples can be obtained frequently at home, they require training of subjects. Maintenance of cold-chain storage may be challenging in warm climates but preservatives can improve specimen quality. SCS have been widely accepted for studies of HSV-2 (reviewed in Barnabas and Celum^5^).

We compared same-day collected CVL and SCS for three genital HIV-1 and HSV-2 outcomes: frequency of detection; viral load; and ability to detect the suppressive effect of acyclovir. We also evaluated the acceptability and feasibility of SCS.

## Methods

We enrolled 67 women co-infected with HIV-1- and HSV-2 in a clinical trial of suppressive acyclovir in Chiang Rai, Thailand (NCT00362596 at www.clinicaltrials.gov and 8). At baseline, median CD4 count was 366 cells/µl, plasma HIV-1 viral load was 4.6 log_10_ copies/ml, and baseline plasma viral load was associated with HIV-1 shedding in SCS.^18^ Women did not receive ART during the trial. They received acyclovir or placebo the first month, no product the second month, and crossed over to placebo or acyclovir the third month. Women were trained in collection of SCS and answered interview questions about acceptability. The study received ethical approval by the Thailand Ministry of Public Health and the Centers for Disease Control and Prevention (CDC).

During the first and third months, women were asked to collect daily genital SCS beginning the day after menstrual bleeding stopped until the next menstrual period began, as described (see Appendix).^18^ Day 1 of menses defined the first day of each month and the last day was defined as the day prior to the start of next menses, as described.^8,18^ Women swabbed the vaginal, vulvar, and perianal areas each morning, inserted the Dacron swab into a tube with ∼150 µl of a proprietary DNA/RNA preservative (Assay Assure^R^, Sierra Molecular Corporation, Sonora, CA) and placed the tubes in coolers containing ice packs and strips to indicate temperatures ≥30°C. Women brought the coolers to on the clinic on days 7, 14, and 21 of the menstrual cycle and were told not to have sex the night before. Swabs were stored at −70°C. On the same day, an additional vaginal swab (for semen detection using the ABAcard p30 kit, Abacus Diagnostics, West Hills, CA) and 10 ml CVL were collected in the clinic as described.^8,18^ After testing for red blood cells (Multistix 8SG, Siemens Healthcare Diagnostics, Tarrytown, NY), 1.2 ml aliquots of CVL were frozen whole for HSV-2 testing. The remaining CVL was spun to separate supernatant (for HIV-1 RNA testing) from cells and stored at −70°C.

Detailed descriptions of processing and nucleic acid extraction are published.^8,18^ For CVL, HIV-1 RNA assayed using 1 or 2 ml of CVL supernatant in an Amplicor Monitor HIV-1 version 1.5 assay (Roche Diagnostic Systems, Branchburg, NJ) using silica-based extraction as described.^8^ HSV-2 DNA was detected and measured using 1 ml of whole CVL in a HSV-2 glycoprotein G gene-targeted TaqMan assay as described.^8,18^ For SCS, an extract was generated using Amplicor reagents, dithioerithretol, and heating as described.^18^ The same extract was used to detect HIV-1 RNA using the Amplicor 1.5 assay and HSV-2 DNA using the TaqMan-based assay. For CVL, viral loads were reported as copies/ml and the lower limits of quantitation (LOQ) and detection (LOD), respectively, were 80 and 40 copies per milliliter for HIV-1 and 50 and 5 copies/ml for HSV-2. For SCS, viral loads were reported as copies/swab and the LOQ and LOD were, 80 and 40 copies per swab for HIV-1 and 400 and 40 copies per swab for HSV-2. HIV-1 results were invalid if there was no amplification of the internal control.

For 67 women, there were 402 possible paired specimens (three samples each from treatment and placebo months). There were 382 available paired specimens that had at least one of the four measurements (detected or not for each virus; viral load for each virus). Of these, three CVL and four SCS had invalid HIV-1 Amplicor assay results, four SCS were excluded due to a high temperature record, and 10 SCS were extracted with a slightly different method. This gave 361 pairs for HIV-1 CVL/SCS comparison and 378 pairs for HSV-2 CVL/SCS comparison. Viral data were categorized as ordinal multinomial outcomes, with non-detectable (below the LOD) and detectable but non-quantifiable (below the LOQ) classified in the first two ordinal categories and subsequent categories defined by increasing logarithm base-10 increments (LOQ–<10^3^, 10^3^– < 10^4^, 10^4^– < 10^5^, ≥ 10^5^ copies/mL).

We compared HIV-1 and HSV-2 detection by calculating an odds ratio of the numbers detected and not detected in SCS and CVL. We also compared proportions of samples with no detectable virus using a Fisher’s exact test. To evaluate the statistical dependence between measures of CVL and SCS, a non-parametric rank correlation coefficient, Spearman’s rho, was computed.

We hypothesized that viral shedding and the estimated acyclovir effects on levels of HIV-1 and HSV-2 were equivalent when measured by SCS or by CVL. To test this, we constructed regression models using generalized estimating equations with an exchangeable working correlation matrix to account for the lack of independence of repeated measurements per subject. In implementing the regression models, we used a multinomial distribution and cumulative logit link to estimate proportional odds ratios and p-values.^19^ A score test was applied to test the assumption of proportional odds. Differences in treatment effect size, as measured from CVL and SCS, were assessed by inclusion of an interaction term (acyclovir treatment by specimen type) in the models. Based on the previous analysis of the suppressive effects of acyclovir on HIV-1 shedding in CVL,^8^ the following covariables were assessed for association or confounding with the four outcome measures and for potential colinearity with each other before including in the regression models: plasma viral load ( < 1000, < 10,000, < 100,000, 100,000 + copies/mL), CVL white blood cell count (<5000, < 10,000, 10,000+), blood CD4 count (<200, < 350, < 500, 500 + cells/mm^3^), genitourinary disease-positivity, bacterial vaginosis, treatment sequence, study week, yeast infection, Trichomonas. Plasma VL and genitourinary disease-positivity were associated with all four outcome measures. CVL white blood cell count and CD4 were associated with the HIV outcome measures. A potential confounding factor was genitourinary disease. Colinearity existed between the explanatory covariables plasma VL and CD4.

## Results

### HIV-1 results: Comparison of CVL and SCS for detection and quantitation of HIV-1

**Figure 1.**
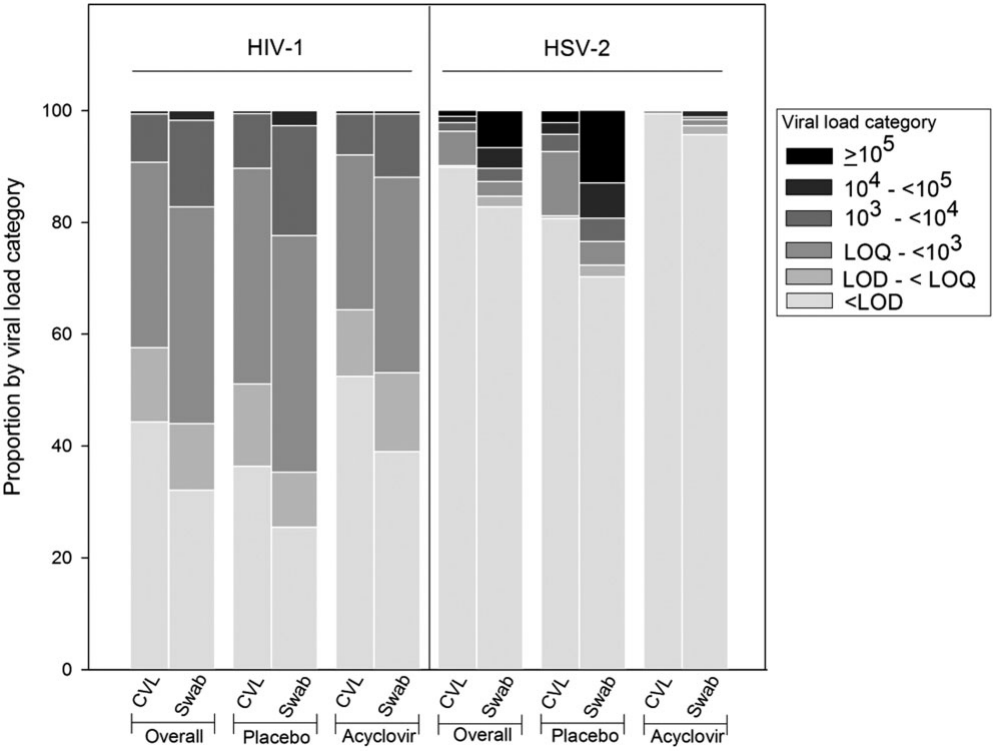
Comparison of collection methods for HIV-1 and HSV-2. Stacked bar graph comparing HIV-1 and HSV-2 data by collection method, in log_10_ viral load categories. Increasing intensity of gray scale indicates higher viral load categories. Data are categories of log_10_ copies/ml for CVL and of copies/swab for genital self-collected swabs (Swab). Data are shown for HIV-1 and HSV-2 either overall or by placebo or treatment arms individually. CVL and swab data are shown side by side for ease of comparison. CVL: cervicovaginal lavage; LOD: limit of detection; LOQ: limit of quantitation.

**Figure 2.**
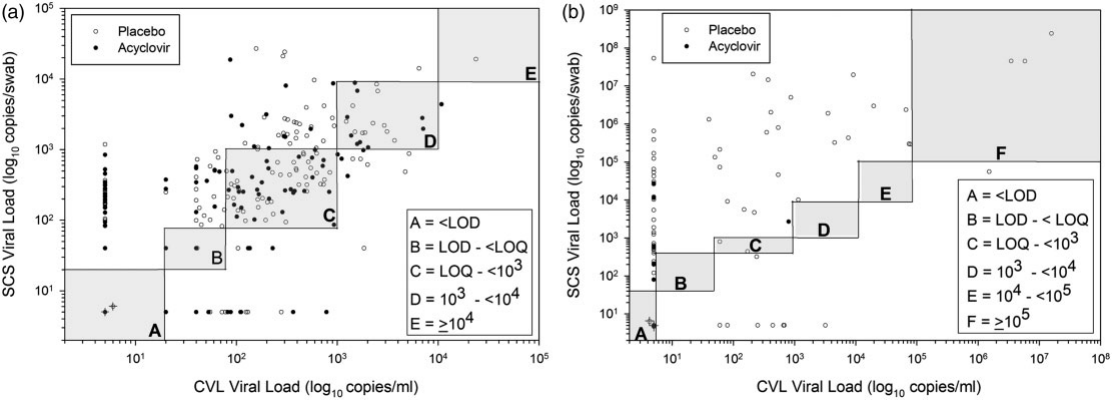
Correlations of viral loads of HIV-1 and HSV-2 in self-collected swabs and cervicovaginal lavage. Data plotted as log_10_ copies/ml for CVL and log_10_ copies/swab for SCS and are shown for HIV-1 (a) and HSV-2 (b). Placebo arm data are in open circles and acyclovir arm data are in closed circles. The shaded boxed areas represent the viral load categories used in the analysis (as in Table 1). Circles with the “cross” in the bottom left box labeled “A” in each graph represent the samples that were below the LOD (36 paired placebo samples and 58 paired acyclovir samples for HIV-1 in Figure 2(a); 128 paired placebo samples and 178 paired placebo samples for HSV-2 in Figure 2(b)). To calculate the number of instances where a sample had detected SCS HIV-1 or HSV-2, but negative CVL values (e.g. dots along *y-*axes) use the first rows of Table 1A and B (<LOD, data in “overall columns”). For HIV-1, this would be CVL #–SCS # (160–116 = 54). Similarly to determine how many in placebo or acyclovir (open and closed circles) perform the same calculation using the data from appropriate columns. Correlations were performed as described. CVL: cervicovaginal lavage; LOD: limit of detection; SCS: self-collected genital swabs.

Of the 361 same-day CVL and SCS samples, a higher proportion of SCS than CVL had detectable HIV-1 RNA values whether analyzed overall or by placebo or acyclovir months (Figure 1, left panels, Figure 2(a) and Table 1A). For example, during the placebo month, when the number of samples with detectable and quantifiable HIV-1 RNA was combined, 74.5% of SCS had detectable HIV-1 shedding compared with 63.6% of CVL (p = 0.001). This pattern of a greater proportion of samples being detected in SCS than in CVL was also noted during the acyclovir month (61.0% and 47.5%, p = 0.001) and overall (67.9% and 55.7%, p < 0.001). Conversely, for all groups (overall or broken down into placebo or treatment month), a greater proportion of CVL than SCS samples were below the detection limit (p < 0.0001, Fisher’s exact test). Detectable values resulting from SCS samples were 1.7 times those of CVL samples.

**Table 1.** Detection and quantitation of HIV-1 and HSV-2 in cervicovaginal lavage and self-collected swabs.

A	HIV-1 CVL			HIV-1 SCS		
	Overall	Placebo	Acyclovir	Overall	Placebo	Acyclovir
	n (%)	n (%)	n (%)	n (%)	n (%)	n (%)
<LOD	160 (44.3)	67 (36.4)	93 (52.5)	116 (32.1)	47 (25.5)	69 (39.0)
Detected <LOQ	48 (13.3)	27 (14.7)	21 (11.9)	43 (11.9)	18 (9.8)	25 (14.1)
LOQ–<10^3^	120 (33.2)	71 (38.6)	49 (27.7)	140 (38.8)	78 (42.4)	62 (35.0)
10^3^–<10^4^	31 (8.6)	18 (9.8)	13 (7.3)	56 (15.5)	36 (19.6)	20 (11.3)
10^4^–<10^5^	2 (0.6)	1 (0.5)	1 (0.6)	6 (1.7)	5 (2.7)	1 (0.6)
B	HSV-2 CVL			HSV-2 SCS		
<LOD	340 (90.0)	155 (80.7)	185 (99.5)	313 (82.8)	135 (70.3)	178 (95.7)
Detected <LOQ	1 (0.3)	1 (0.5)	0 (0)	7 (1.9)	4 (2.1)	3 (1.6)
LOQ–<10^3^	23 (6.1)	22 (11.5)	1 (0.5)	10 (2.7)	8 (4.2)	2 (1.1)
10^3^–<10^4^	6 (1.6)	6 (3.1)	0 (0.0)	9 (2.4)	8 (4.2)	1 (0.5)
10^4^–<10^5^	4 (1.1)	4 (2.1)	0 (0.0)	14 (3.7)	12 (6.3)	2 (1.1)
≥10^5^	4 (1.1)	4 (2.1)	0 (0.0)	25 (6.6)	25 (13.0)	0 (0.0)

There were 361 pairs of CVL and SCS with same-day HIV-1 RNA results (A) and 378 pairs of CVL and SCS with same-day HSV-2 results (B). The proportions of samples with undetectable virus is indicated as <LOD (level of detection). The proportions above or below the LOQ (limit of quantitation) for each assay or sample type, as stated in the ‘Methods’ section are indicated as < or >LOQ. Values are reported as log_10_ copies/ml for CVL or copies/swab for SCS. No values ≥10^5^ were observed for HIV-1.

CVL: cervicovaginal lavage; SCS: self-collected swabs; LOD: limit of detection; LOQ: limit of quantitation.

The figures and Table 1A also show that overall, in placebo and acyclovir months, SCS appeared to have a greater proportion of samples represented in any viral load category than CVL. When analyzing ordered categories of VL in multinomial logistic regression, higher values were measured for SCS relative to CVL (p < 0.001).

To measure the strength of the correlation between the HIV-1 RNA levels in SCS and CVL, non-parametric rank correlations were calculated using the categorized data. There was good correlation (as shown in Figure 2(a)) between the SCS and CVL values, whether overall (Spearman rho, 0.69), during the acyclovir month (Spearman rho, 0.67) or during the placebo month (Spearman rho, 0.67).

Figure 2(a) also shows the frequency of measures in which SCS had detectable HIV-1 RNA and the corresponding CVL had no detectable HIV-1 RNA (data points near *y*-axis) or vice versa (data points near *x*-axis). There are many times when CVL samples had no detected HIV-1 RNA, but same day SCS had detectable HIV-1 RNA with up to several thousand copies per swab. Conversely, there were a few times when CVL samples had detected HIV-1 RNA, but it was not detected in the same day SCS. These differences could not be explained by presence of semen or blood in the samples (data not shown).

Multinomial regression was implemented to evaluate reductions in HIV-1 viral loads in the acyclovir treatment month compared with the control, using the ordered categories of viral load. This approach was selected because so many values were below the LOD or LOQ (Table 1). Significant reductions in HIV-1 RNA in SCS were observed in implementation of univariate and multivariate models (Table 2). In the multivariate model, the proportional odds ratios for these reductions were 0.47 for CVL and 0.42 for SCS; there were no differences in the estimated acyclovir effect on HIV-1 virus loads as measured by SCS compared with CVL (p = 0.20).

**Table 2.** Impact of acyclovir on HIV and HSV-2 as detected by self-collected swabs and cervicovaginal lavage.

Model outcome	Effect	Single variable model Proportional odds ratio (95% CL)	Single variable Model p-value	Multivariable model Proportional odds ratio (95% CL)^a^	Multivariable model p-value
CVL-HIV-1	Acyclovir	0.56 (0.42, 0.75)	<0.0001	0.47 (0.31, 0.71)	<0.001
SCS-HIV-1	Acyclovir	0.49 (0.36, 0.68)	<0.0001	0.42 (0.27, 0.65)	<0.0001
CVL-HSV-2	Acyclovir	0.02 (<0.01, 0.17)	<0.0001	0.03 (<0.01, 0.19)	<0.001
SCS-HSV-2	Acyclovir	0.10 (0.04, 0.28)	<0.0001	0.10 (0.04, 0.29)	<0.0001

a Besides acyclovir, the HIV-1 multivariable models included covariables for plasma viral load, CVL white blood cell count, genitourinary disease positivity, and the HSV-2 multivariable models included covariables for plasma viral load and genitourinary disease positivity. Acyclovir effects are interpreted as odds of higher ordered virus loads per the ordered categories described in Table 1. An assumption of proportional odds was tested as described in the ‘Methods’ section. Model terms testing whether the acyclovir effect was different by CVL vs. SCS sample collection method (two-way interaction effect) were not significant for HIV-1 (p = 0.20) or for HSV-2 (p = 0.33).

CVL: cervicovaginal lavage; SCS: self-collected swabs.

### HSV-2 results: Comparison of CVL and SCS for detection and quantitation of HSV-2

Among the 378 same-days samples of HSV-2 DNA, fewer SCS samples than CVL samples were below the LOD (Figures 1 and 2(b), Table 1, p < 0.0001); 29.7% of SCS had detectable HSV-2 DNA shedding compared with 19.3% of CVL (p < 0.001) in the placebo arm. Higher proportions of detectable viral load values for SCS were also observed in the acyclovir arm (4.3% vs. 0.5% for CVL, p = 0.03) and overall (17.2% vs. 10.0% for CVL, p < 0.001). Detectable values resulting from SCS samples were 1.9 times those of CVL samples.

As observed for HIV-1, some SCS samples had measurable HSV-2, even though the same day CVL sample was negative (Figure 2(b)); similarly, there appeared to be no association with semen or blood.

When the ordered categories (Figure 2(b) and Table 1) were analyzed using multinomial regression, we found that higher values of viral load were measured for SCS relative to CVL (p < 0.001). Significant (p < 0.0001) differences between the proportion of undetected samples comparing SCS and CVL were observed for overall and placebo. The difference between the proportion undetected in the acyclovir month comparing SCS and CVL was minimally significant (p < 0.043).

To measure the strength of the relationship between the HSV-2 DNA levels in SCS with those detected in CVL, non-parametric rank correlations were calculated using the categorized data. There was good correlation between the values generated from both sample types overall (Spearman rho, 0.66) and in the placebo group (Spearman rho, 0.61) (Figure 2(b)). The correlation was weak in the treatment group (Spearman rho, 0.36) possibly due to the suppressive effect of treatment and the large proportion with undetectable DNA levels.

To compare the suppressive effect of acyclovir on HSV-2 as detected by SCS and CVL, the ordered categories of viral load were modeled as described for HIV-1. In multivariate modeling, the proportional odds ratio was 0.03 for CVL and 0.10 for SCS (Table 2); there was no difference in the estimated acyclovir effect on HSV-2 virus loads as measured by CVL or SCS (p = 0.33).

### Acceptability and reverse cold chain

Most (97%) women found it easy or somewhat easy to collect the swabs, most (70%) found swab collection to be comfortable and most (76%) also found it easy to change the cooler ice packs twice daily. Only two women found swab collection to be somewhat difficult. Most (97%) women found it easy to keep the swabs or cooler private. When the temperature records of all 2630 daily swabs were reviewed, only 25 (1%) SCS needed to be excluded because of high temperature.

## Discussion

In this study, we found SCS to be a sensitive measure of HIV-1 and HSV-2 and SCS virus loads positively correlated with those of CVL. Detectable values resulting from SCS were 1.7 and 1.9 times those of CVL for HIV-1 and HSV-2, respectively. SCS could measure acyclovir-associated reductions in HIV-1 and HSV-2 virus loads equally well as CVL. To our knowledge, our study is the first to compare SCS and CVL for detection of both pathogens. Since HIV-1 and HSV-2 are frequent co-infections, whose shedding rates vary in time, use of SCS rather than CVL has many advantages. SCS can be collected frequently. This can be useful for clinical trials, but also may improve understanding of these pathogens. For example, analysis of HIV-1 shedding by menstrual cycle phase using SCS data from women from this study, showed a nadir of shedding in the peri-ovulatory period.^12^ Moreover, having more samples and repeated measures increases the precision of both HSV-2 and HIV-1 shedding estimates.^20,21^ Use of SCS can reduce the number of clinic visits and the need for trained providers. In our trial, women followed instructions carefully and few swabs were discarded because of high temperature readings. Detection of HIV-1 and HSV-2 in one sample also increases laboratory efficiency.

Strengths of our study include that CVL and SCS were collected on the same day, and women were shedding both viruses frequently enough to compare even when shedding rates were reduced due to the suppressive effect of acyclovir. The sample size allowed a statistical approach using ordered viral load categories when many samples had undetectable or unquantifiable results. When the data were analyzed as continuous quantitative data or in a binomial approach based on detection or non-detection of virus, we saw similar correlations between SCS and CVL levels and similar acyclovir-associated reductions in virus load in SCS compared with CVL (data not shown). The significant reductions in CVL viral shedding in this analysis were consistent with what was reported using another statistical approach.^8^

Multiple factors could explain why SCS performed better than CVL in this study including specimen type and collection method, preservative, storage and extraction methods, and analytic approaches. Our study was not designed to address these issues, but we considered them when interpreting our findings.

More virus may have been collected by SCS due to the different mode of collection (vaginal, vulvar, and peri-anal areas) compared with CVL (vaginal and cervical areas). For HSV-2, a swab study in women showed that much shedding occurs peri-anally.^22^ For HIV-1, our data suggest that SCS, as used in this study, may provide an advantage by sampling additional sites of HIV-1 shedding. While we did not evaluate these anatomic sites separately, swabs from genital ulcers in HIV-1 and HSV-2 co-infected men are HIV-1 RNA positive^23^ suggesting that the sampling of extra-vaginal areas in women with HSV-2 may detect additional HIV-1 that may be transmissible. Caution should be used extrapolating our observations to other HIV-1/HSV-2 co-infected women.

SCS were whole samples containing cellular and non-cellular fractions, and while whole CVL were used for HSV-2 assays, CVL supernatants were used for HIV-1, potentially underestimating the amount of HIV-1 in CVL. The impact of this is likely to have been small: the larger sample volume of CVL (2 ml compared with the approximately 150 µl eluted from the swab) could have overcome this difference. We also did not examine whether the sequence of swab and same day CVL collection mattered. A new study comparing swabs with whole CVL and evaluating specimen collection sequence could provide relevant information.

Swab type, DNA/RNA preservative, storage and laboratory methods could have impacted the data. While the SCS were Dacron in a DNA/RNA preservative and subject to reverse cold chain storage for a week, CVL were frozen immediately with no preservative. Future studies could address the effect of each of these factors. For example, women can self-collect CVL.^24^ In our study, extraction methods differed for SCS and CVL. The use of the Amplicor-based procedure for SCS could have resulted in more proviral HIV-1 DNA being extracted and amplified, potentially resulting in the swabs having higher values. Another study, using the same extraction protocol for both samples could determine the relevance of this step as well as address the possibility of PCR inhibitors.

Our findings, that SCS detected and quantified HIV-1 and HSV-2 more frequently than CVL and could detect acyclovir-induced suppression of HIV-1 and HSV-2, suggest that SCS can be used in studies to advance efforts to control HIV-1, particularly in settings where there is co-infection with HSV-2.

## Appendix 1 (section from Forhan et al.^18^ on SCS collection)

Genital SCS were collected daily from participants during the intervention and placebo months of the study. Specifically, during the clinical trial, each participant was asked to collect an SCS daily beginning the day after menstrual bleeding stopped through the day before the next menstrual period began. For women with no menses, collection of SCS began on a selected day and continued for 28 days. At the enrollment visit, each participant received instructions about the SCS specimen collection, and how to store the specimen in a cooler using twice daily ice pack changes. The participant demonstrated the collection technique to research staff. To collect a genital SCS specimen, the participant used a single Dacron swab which she sequentially swabbed over: (a) the vaginal area (approximately two inches into the vaginal vault), (b) the vulvar area, and (c) the perianal region, for a total of 15 seconds. The participant then inserted the swab into a labeled tube containing a sponge impregnated with approximately 150 µl of Assay Assure® (formerly Genelock, Sierra Molecular Corporation, Sonora, CA, USA) that included a proprietary DNA/RNA preservation solution, capped the tube, and placed it into a cooler which contained frozen ice packs and temperature monitoring strips (3M Monitor Mark, Cold Chain Technologies, Holliston, MA, USA).

To maintain the temperature within the cooler between approximately 4–17°C, each participant changed the cooler ice packs twice daily. A pilot evaluation conducted by the laboratory demonstrated that swabs could be maintained at 4–17°C using these methods, at ambient temperature in Thailand. A temperature monitoring strip placed in the cooler noted if temperatures exceeded 30°C. Participants transported coolers (containing swabs) weekly to their study visit. Research staff collected the swabs, recorded information from the temperature monitoring strip within the cooler, and, within two hours of delivery, stored the swabs at −70°C. Swabs were then shipped frozen to the laboratory for evaluation.
